# Carbon nanotube/Co_3_O_4 _composite for air electrode of lithium-air battery

**DOI:** 10.1186/1556-276X-7-28

**Published:** 2012-01-05

**Authors:** Taek Han Yoon, Yong Joon Park

**Affiliations:** 1 Department of Advanced Materials Engineering, Kyonggi University, Suwon, Gyeonggi-do, 443-760, Republic of Korea

**Keywords:** composites, nanostructures, chemical synthesis, electrochemical properties

## Abstract

A carbon nanotube [CNT]/Co_3_O_4 _composite is introduced as a catalyst for the air electrode of lithium-air [Li/air] batteries. Co_3_O_4 _nanoparticles are successfully attached to the sidewall of the CNT by a hydrothermal method. A high discharge capacity and a low overvoltage indicate that the CNT/Co_3_O_4 _composite is a very promising catalyst for the air electrode of Li/air batteries.

## Introduction

Lithium-based batteries have found new applications in technologies such as electric vehicles, plug-in hybrid electric vehicles, robots, and electric power storing systems. However, commercial lithium-ion batteries still do not offer enough energy density for these high power consumption devices although extensive research has been conducted to increase charge-storage capability [1-4]. The energy storage of lithium-based batteries can be remarkably enhanced by a new approach, lithium-air [Li/air] batteries [5-8]. A Li/air battery consists of a Li metal anode and an air electrode containing a catalyst. Oxygen accessed from the environment is reduced catalytically on the air electrode surface to form anions, which then react with lithium cations supplied by the anode on the air electrode surface during the discharge process [9,10]. Owing to light and unlimited cathode active material (oxygen), Li/air batteries have a much larger theoretical specific energy (11,400 Wh·kg^-1 ^excluding oxygen) than any other rechargeable battery system including lithium-ion batteries. In this work, carbon nanotubes [CNTs] and nanosized Co_3_O_4 _were successfully composited to catalyze the anion formation in the air electrode of Li/air batteries. The CNT serves to support the catalyst and provides a surface for the redox reaction to occur. Co_3_O_4 _has generated extensive interest as a promising catalyst in various fields [11,12]. Co_3_O_4 _nanoparticles composited with CNT are expected to show excellent catalytic activity owing to their nanoscale size and large surface area.

### Experimental details

First, 0.5 g of purified multiwall CNTs [MWCNTs] was dispersed in 50 ml of 1 wt.% cetyltrimethylammonium bromide aqueous solution for 30 min, which was followed by centrifuging and washing. Then, the MWCNTs were mixed with 50 ml of a 1 wt.% aqueous solution of poly sodium 4-styrenesulfonate [PSS] and stored for 12 h. After removing excess PSS, the MWCNTs were dispersed in 40 ml ethylene glycol [EG] by sonication for 30 min. Then, 0.5 g of Co_3_O_4 _nanoparticles (Sigma-Aldrich, St. Louis, MO, USA) was dispersed in 40 ml of functionalized MWCNT EG solution. Next, 3.0 g of NaAc (C_2_H_4_NaO_2_) and 1.0 g of polyethylene glycol were added with constant stirring for 30 min. The solution was then transferred to a Teflon-lined stainless steel autoclave with 100 ml capacity and kept at 200°C for 14 h. The black product was washed and dried at 90°C. X-ray diffraction [XRD] patterns of the powder were measured using a Rigaku X-ray diffractometer (Rigaku Corporation, Tokyo, Japan). The microstructure of the powder was observed by field-emission scanning electron microscopy (JEOL-JSM 6500F; JEOL, Ltd., Akishima, Tokyo, Japan) and field-emission transmission electron microscopy (JEOL-JEM 2100F; JEOL, Ltd., Akishima, Tokyo, Japan). The electrochemical performance of the air electrode containing CNT/Co_3_O_4 _composite was examined using a modified Swagelok cell (Swagelok Company, Solon, OH, US) consisting of a cathode, metallic lithium anode, glass fiber separator, and an electrolyte of 1 M LiTFSi in EC/PC (1:1 vol.%). The cathode contained carbon (Ketjen black), catalyst (CNT/Co_3_O_4 _composite), and binder (polyvinylidene fluoride). The weight ratio of the CNT/Co_3_O_4 _composite to carbon was adjusted to 80:20, and 10 wt.% of the binder for the total electrode was used. The cells were subjected to galvanostatic cycling using a WonAtech (WBCS 3000; WonAtech, Seoul, South Korea) charge-discharge system. Experiments were carried out in 1 atm of O_2 _using an air chamber.

## Results and discussion

**Figure 1:**
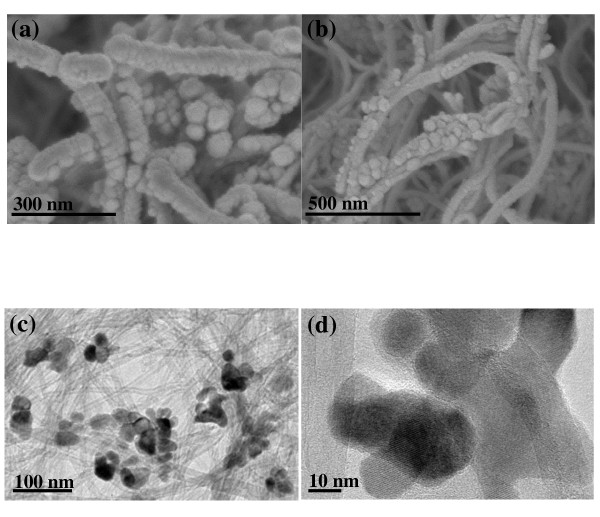
**Shape and morphology of the CNT/Co_3_O_4 _composite**. (**a**, **b**) SEM images of the composite. (**c**) TEM image of the composite. (**d**) TEM image of the Co_3_O_4 _nanoparticle of the composite.

**Figure 2:**
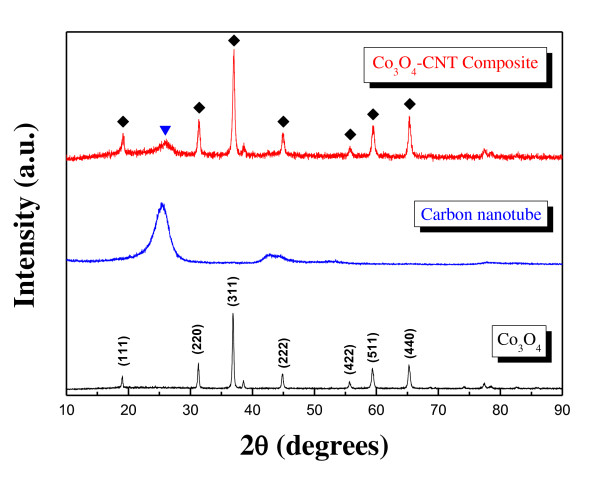
**XRD patterns of CNT/Co_3_O_4 _composite, CNT, and typical Co_3_O_4 _powder**.

The shape and morphology of the CNT/Co_3_O_4 _composite was observed using scanning electron microscopy [SEM] and transmission electron microscopy [TEM], as shown in Figure 1. In the SEM images, it was observed that Co_3_O_4 _nanoparticles with spherical shapes were attached to the sidewalls of the CNT. As shown in the TEM image in Figure 1c, the distribution of the Co_3_O_4 _nanoparticles was not perfectly uniform, but virtually no free nanoparticles were found in any of the images. The size of the Co_3_O_4 _nanoparticles was 20 to 30 nm. The formation of the CNT/Co_3_O_4 _composite was also confirmed by XRD analysis. The patterns from which are shown in Figure 2 (top). The crystalline peaks can be clearly indexed to a typical Co_3_O_4 _crystalline phase with a spinel structure. The broad peak located at approximately 2*θ *= 23.5° is characteristic of a CNT. The weight ratio of the CNT to the Co_3_O_4 _crystalline phase was 60:40, as determined by inductively coupled plasma atomic emission spectroscopy analysis.

**Figure 3:**
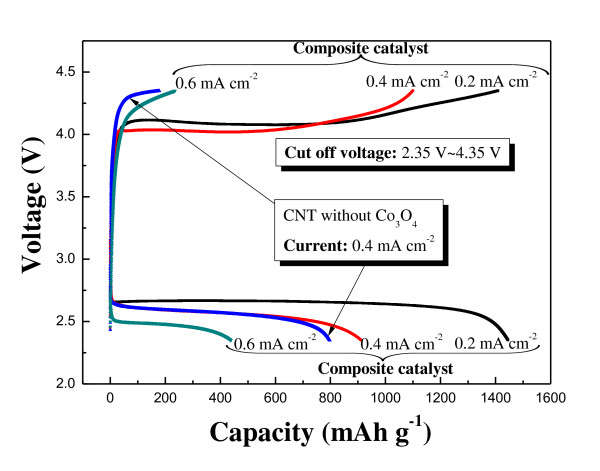
**Initial charge and discharge profiles**. The electrodes containing the CNT/Co_3_O_4 _composite and only CNT are at constant current densities of 0.2, 0.4, and 0.6 mA·cm^-2 ^in the voltage range of 4.35 to 2.35 V.

To investigate the electrochemical properties of the composite as a catalyst for the air electrode, the test cell containing the composite was characterized. An air electrode containing only CNT without Co_3_O_4_, which had the same catalyst (CNT) to carbon to binder ratio, was also prepared and characterized for comparison of the electrochemical properties to those of the composite. Figure 3 shows the initial voltage profile of the electrodes at constant current densities of 0.2, 0.4, and 0.6 mA·cm^-2 ^in the voltage range of 4.35 to 2.35 V (30°C). The capacity shown in Figure 3 was based on the total electrode mass (CNT/Co_3_O_4 _composite + carbon + binder), which may be reasonable to present the storage ability of energy as rechargeable batteries. The average charge voltage of the electrode containing the composite was around 4.1 to 4.2 V at current densities of 0.2 and 0.4 mA·cm^-2^. In contrast, the charge voltage of the electrode containing only the CNT showed over 4.3 V at a current density of 0.4 mA·cm^-2^. The high charge voltage indicates high overvoltage, so the composite electrode appeared to have lower overvoltage than the electrode containing only the CNT. This implies that the CNT/Co_3_O_4 _composite is a good catalyst for reducing the overvoltage during the charge process. As expected, the average charge voltage increased as the current density increased to 0.6 mA·cm^-2^, which is still lower than that of the CNT electrode.

The discharge capacity of the air electrode containing the composite catalyst reached approximately 1,450 mAh·g^-1 ^at a current density of 0.2 mA·cm^-2^. As the current density was increased to 0.4 and 0.6 mA·cm^-2^, the discharge capacity of the electrode decreased to approximately 950 and 450 mAh·g^-1^, respectively. This discharge capacity of the air electrode is much higher than that of typical cathode materials used in lithium-ion batteries. In general, the specific discharge capacity of the cathode, which is composed of intercalation oxide, carbon, and binder, for lithium-ion cells is just 120 to 170 mAh·g^-1^. Furthermore, the air electrode containing the CNT/Co_3_O_4 _composite demonstrates superior discharge capacity compared to those previously reported (600 to 800 mAh·g^-1 ^based on the total electrode mass) for the air electrode containing oxide catalysts [5,6]. This may be attributed to the large reaction surface area provided by the CNT matrix supporting the nanosized Co_3_O_4 _particles. The discharge process of the air electrode is terminated when the total catalytic active sites are blocked by reaction products [10]. The Co_3_O_4 _particles distributed in the walls of the CNT may provide abundant catalytic active sites, which could extend both the discharge process and the capacity. Moreover, the stable contact between Co_3_O_4 _and the CNT will facilitate electron conduction during reaction. The air electrode containing only the CNT showed a discharge capacity that is considerable but smaller than that of the composite electrode. In addition, the charge capacity of the CNT electrode was very small, indicating poor reversibility.

**Figure 4:**
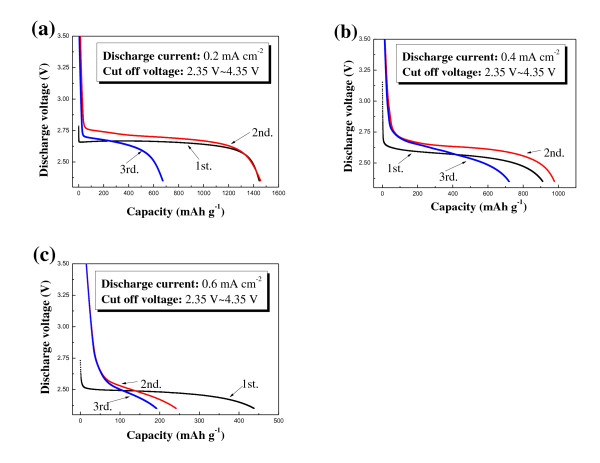
**Discharge profiles of the air electrodes containing the CNT/Co_3_O_4 _composite for the initial three cycles**. At a current density of (**a**) 0.2 mA·cm^-2^, (**b**) 0.4 mA·cm^-2^, and (**c**) 0.6 mA·cm^-2^. (**d**) Cyclic performance of the electrodes (current density, 0.4 mA·cm^-2^).

Figures 4a to c show the discharge profiles of the air electrode containing the CNT/Co_3_O_4 _composite for the initial three cycles at constant current densities of 0.2, 0.4, and 0.6 mA·cm^-2 ^in the voltage range of 4.35 to 2.35 V. The discharge capacity and the voltage profile changed significantly from their initial values, indicating that the reaction was not fully reversible. As shown in Figure 4d, the cyclic performance of the composite electrode was not sufficient for a rechargeable electrode although it was much more enhanced than that of the CNT electrode. Actually, capacity fading has been a typical feature of all previous results about the air electrode [6,9,10]. It has been established that insoluble reaction products are formed during the discharge process, which accumulate during cycling, block the active catalytic sites, and decrease the capacity of the air electrode [9,10]. The reaction products may be composed of lithium oxides (Li_2_O_2 _and/or Li_2_O) formed from the reaction between Li ions and oxygen. Carbon products such as C_3_H_6_(LiOCO_2_)_2_, Li_2_CO_3_, LiHCO_2_, and LiCH_3_CO_2 _are produced from the decomposition of the carbonate electrolyte and reaction with Li ions during cycling [13,14]. To avoid capacity fading and obtain more stable cyclic performance of the air electrode, it may be necessary to control reaction products and decomposition of the electrolyte.

## Conclusions

A CNT/Co_3_O_4 _composite was successfully fabricated for use in the air electrodes of Li/air batteries. Nanosized Co_3_O_4 _particles (20 to 30 nm) were attached to the outer surface of the CNT. The air electrode containing the CNT/Co_3_O_4 _composite exhibited a high discharge capacity and low overvoltage during the charge-discharge process, which indicates that the composite is potentially a good catalyst for the air electrode.

## Abbreviations

CNT: carbon nanotubes; EG: ethylene glycol; MWCNTs: multiwall carbon nanotubes; PSS: poly sodium 4-styrenesulfonate; SEM: scanning electron microscopy; TEM: transmission electron microscopy; XRD: X-ray diffraction.

## Competing interests

The authors declare that they have no competing interests.

## Authors' contributions

TH performed the synthesis and characterization in this study. YJ gave the advice and guided the experiment. Both authors read and approved the final manuscript.
